# Exploring the Impact of Small Group Teaching and Case-Based Learning on Optometry Students’ Binocular Vision Clinical Knowledge

**DOI:** 10.22599/bioj.486

**Published:** 2025-08-25

**Authors:** Sirawit Ketchan, Ketan Parmar, Catherine Porter

**Affiliations:** 1 The University of Manchester, UK

**Keywords:** optometric education, binocular vision, case-based learning, small group teaching

## Abstract

**Introduction:**

Small group teaching is widely used in healthcare education. Few studies have specifically focused on small group teaching in optometry. This study investigated the effects of small group clinical teaching combined with case-based learning (CBL) on final-year optometry students’ knowledge in the assessment, diagnosis and management of accommodation and convergence anomalies.

**Methods:**

Students took part in a tutorial session involving the assessment of accommodation and convergence. A total of 89 final-year optometry students were randomly divided into two groups (control and intervention) prior to attending a binocular vision tutorial. Before undergoing the tutorial, the control group completed 20 multiple-choice questions (sets A and B), whilst the intervention group answered 10 questions (set A) before and the remaining 10 questions (set B) after the tutorial. Non-parametric statistics were employed to investigate the differences in Set A and B scores within and between the groups.

**Results:**

There was no significant difference in set A scores between the control (48.18%, ±17.16) and intervention (43.81%, ±13.43) groups (p = 0.27). The intervention group had significantly higher set B scores (60.71%, ±17.02) than the control group (50.68%, ±17.84) (p = 0.01).

**Conclusion:**

Small group teaching combined with CBL significantly enhances optometry students’ knowledge of the diagnosis and management of accommodation and convergence anomalies. This teaching and evaluation methodology has the potential to be applied across all healthcare disciplines.

## Introduction

Small group teaching is a pedagogical approach widely used in medical education. It focuses on actively engaging learners and encouraging their participation (Crosby, 1996), has been shown to improve healthcare students’ knowledge of cardiovascular physiology and pharmacology (Brandl, Schneid and Laiken, 2022), and is perceived as a beneficial learning experience by students and educators alike (Nayak *et al*., 2021; Zeri *et al*., 2020). Zeri *et al*. (2020) reported optometry students perceive that group work improves problem solving and clinical decision making. Edmunds and Brown (2010) reported it is common in the UK to have groups of 6–8 students for small group learning and teaching activities. A recent meta-analysis by Huang *et al*. (2019), comparing effective teaching in clinical education, showed that small group teaching had significant positive impacts on student learning associated with ‘Teaching Learning Factors’ (aspects that affect teaching or learning processes) and should be advocated in clinical education.

Clinical knowledge and skills are integral to healthcare professionals’ education. In healthcare undergraduate education, a growing trend in teaching methodology is the use of case-based learning (CBL) (Jesus, Gomes and Cruz, 2012; Romero, Eriksen and Haworth, 2004). Learning in clinical subjects involves understanding the diagnosis and treatment of patients presenting with health conditions. CBL in a healthcare context involves presenting students with clinical scenarios, encouraging them to diagnose the health condition and develop holistic management strategies. This requires them to link theory to practice by applying their subject knowledge to an authentic patient case (Thistlethwaite *et al*., 2012). Studying anonymised real patient scenarios can greatly benefit healthcare students (Korniichuk *et al*., 2021) allowing them to engage with real-world patient issues that they might not otherwise encounter regularly in a clinical environment.

The General Optical Council (GOC) set competency standards (GOC, 2015) for undergraduate optometry students, some of which relate to practical skills in binocular vision. Therefore, an understanding of the diagnosis and management of binocular vision disorders is a mandatory component of UK optometry programmes. Consequently, to meet the regulator’s standards, optometry schools must design and implement effective teaching methods. At The University of Manchester, final year undergraduate optometry students are taught practical binocular vision skills in small group clinical sessions, which are combined with CBL. The authors set out to evaluate if this teaching methodology improves undergraduate optometry students’ knowledge of the diagnosis and management of accommodative and convergence anomalies.

## Methods

This was a prospective, experimental, quantitative experiment designed to investigate the effect of small group clinical teaching and CBL on student knowledge of the diagnosis and management of accommodation and convergence anomalies. There were 89 final (third) year optometry students at The University of Manchester in the 2023/24 academic year. Since this study was an evaluation of teaching, The University of Manchester exempted formal ethical approval. One author (CP) anonymously retrieved all data from the Virtual Learning Environment, and no personal identifiable information was collected or stored.

In developing the case-based teaching, specific design principles were implemented. Newble and Cannon (2001) state that small group teaching should contain ‘active participation, face-to-face contact, and purposeful activity.’ Binocular vision teaching at The University of Manchester uses the principle of constructive alignment to design teaching and learning activities (Biggs and Tang, 2011). The process began by identifying learning outcomes, which were then used to create a practical skills session and a CBL scenario. The patient in the case was anonymised from one of the author’s (CP) clinical cases.

Students worked in pairs for the practical session. They assessed accommodation (amplitude, facility and accuracy) as well as convergence (jump and smooth pursuit) functions. Students also practised a range of common vision therapy exercises used to improve accommodation (e.g., the Hart Chart) and convergence (e.g., Brock String and Dot Card) in optometric practice. The students then worked in groups of five or six to review a clinical case scenario of a child with an accommodation anomaly and devise a management plan. After this, a whole group discussion was facilitated by two academic staff (CP and KP). The duration of the teaching session was 2.5 hours and delivered by two academic staff (CP and KP). All the teaching materials are provided in Appendix 1.

**Figure 1:**
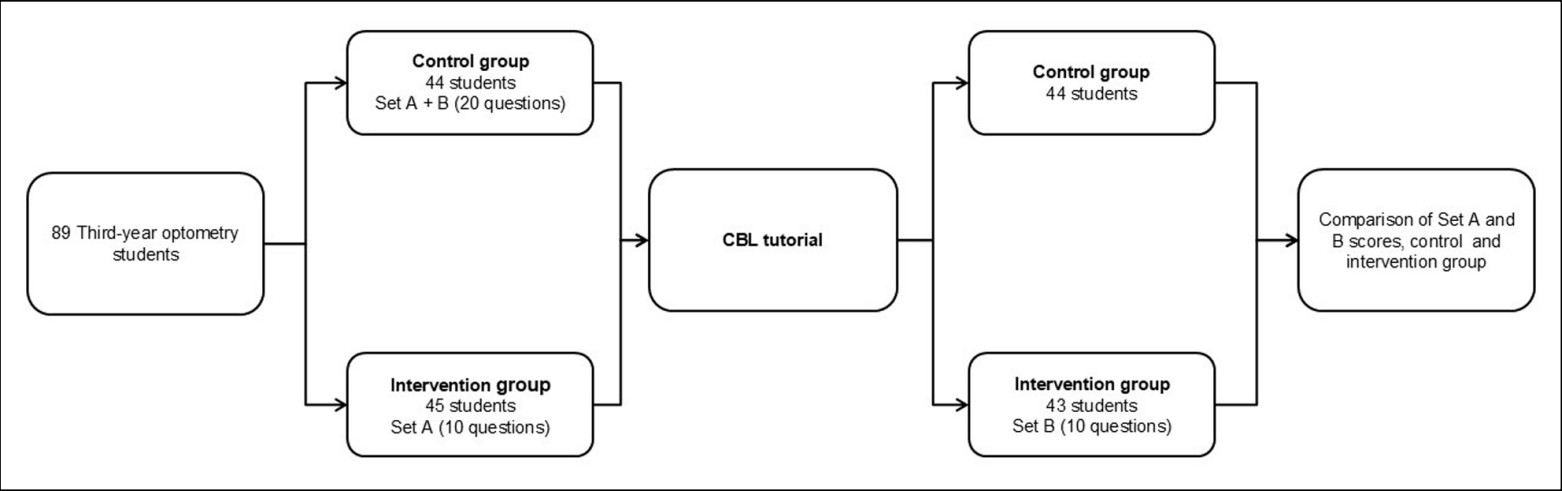
Diagram showing numbers of students in the control and intervention group and how the teaching session and assessment was organised.

**Applying the evaluation method**. Before the practical session, students were randomly assigned into either a control or intervention group. The intervention group completed 10 MCQs (set A) immediately before the session and 10 MCQs (set B) after. The control group completed all 20 MCQs (sets A and B) just before the practical session. Questions were presented individually in a random sequence, and students were unable to revisit previously answered MCQs. Scores were withheld until the teaching session concluded. The students attended the practical sessions between October and November 2023. Figure 1 shows how the students were allocated to groups, and the non-attendance in each group.

**Developing the MCQs**. In the 2022–23 academic year, a set of 20 multiple-choice questions (MCQs) (Appendix 2) was created and evaluated to assess their effectiveness in distinguishing between high-performing and low-performing students. To establish content validity and verify the accuracy of the answer key, two academic optometrists reviewed the questions. The evaluation process involved individual assessments followed by group discussions to resolve any remaining disagreements. This key check ensured that the answers to the MCQs were both unambiguous and correct. Construct validity was determined through item difficulty analysis and discrimination ability (Considine, Botti and Thomas, 2005). The learning management system’s built-in software analysis was used to examine the questions for discrimination and difficulty. Only questions with a discrimination index of 0.21 or higher were selected for use (Date *et al*., 2019). In the present study, 10 of these MCQs were used for the ‘pre-test’ (set A) and 10 were used for the ‘post-test’ (set B). There was an equal number of easy/difficult questions in each subset.

### Data analysis

All analyses were carried out using SPSS version 29. The Shapiro-Wilk test confirmed data were not normally distributed. Therefore, the Mann-Whitney U test and Wilcoxon signed-rank tests were used to look for statistical differences in the MCQ scores of the two groups. Using this approach, any improvement in scores post-session could be attributed to student engagement in the tutorial. This novel method has been used previously to evaluate an Interprofessional Education workshop (Porter and Parmar, 2025).

## Results

A total of 87 (out of a possible 89) optometry students attended the small group teaching CBL session. Of these, only one student did not complete the post-session questions, and their results were excluded from the analysis. This left 86 optometry students (97% of the cohort) with complete data sets.

**Figure 2:**
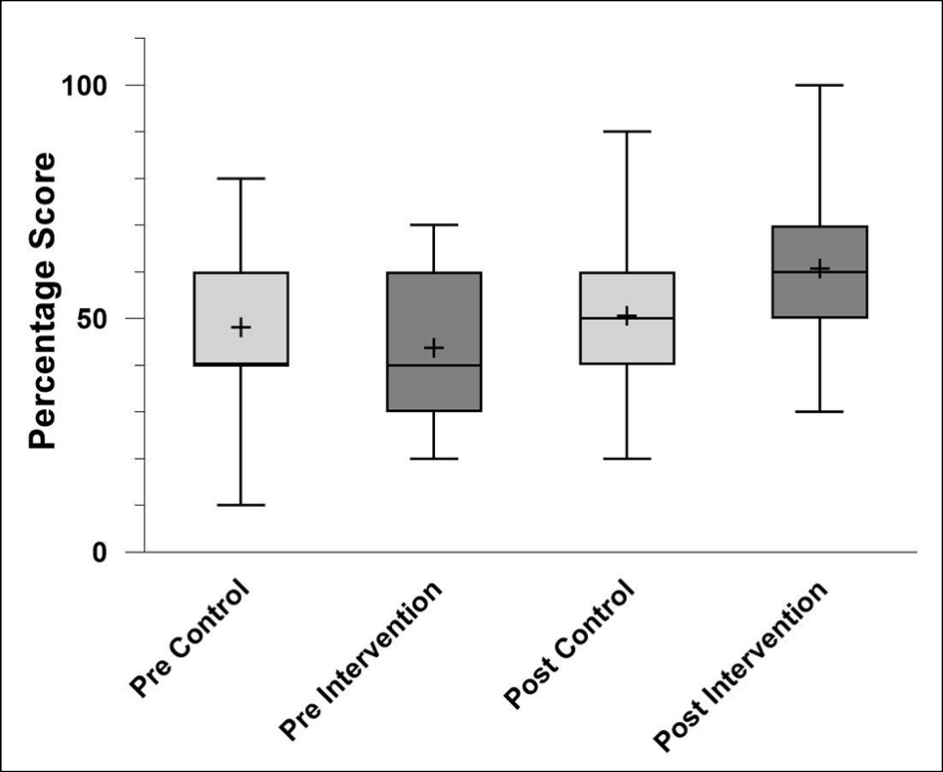
Box and Whiskers plot of pre- and post-scores of the control and intervention groups. The plot displays the distribution of scores, with the central line indicating the median and the plus sign representing the mean. Error bars denote the range.

The average set A and set B MCQ scores for the two groups are shown in Figure 2.

The control and intervention groups did not significantly differ in their set A scores (p = 0.27), both completed before the teaching session. The intervention group (n = 42) had a mean score of 43.81% (±13.43). The control group (n = 44) had a mean score of 48.18% (±17.16).

The average set B scores of the control group (completed pre-session) were 50.68% (±17.84) and the intervention group (completed post-session) 60.71% (±17.02%). This was a statistically significant difference (p = 0.01).

There was no significant difference between the set A and set B scores for the control group (p = 0.31). However, the difference in the set A and set B scores for the intervention group was statistically significant (p < 0.01).

**Figure 3:**
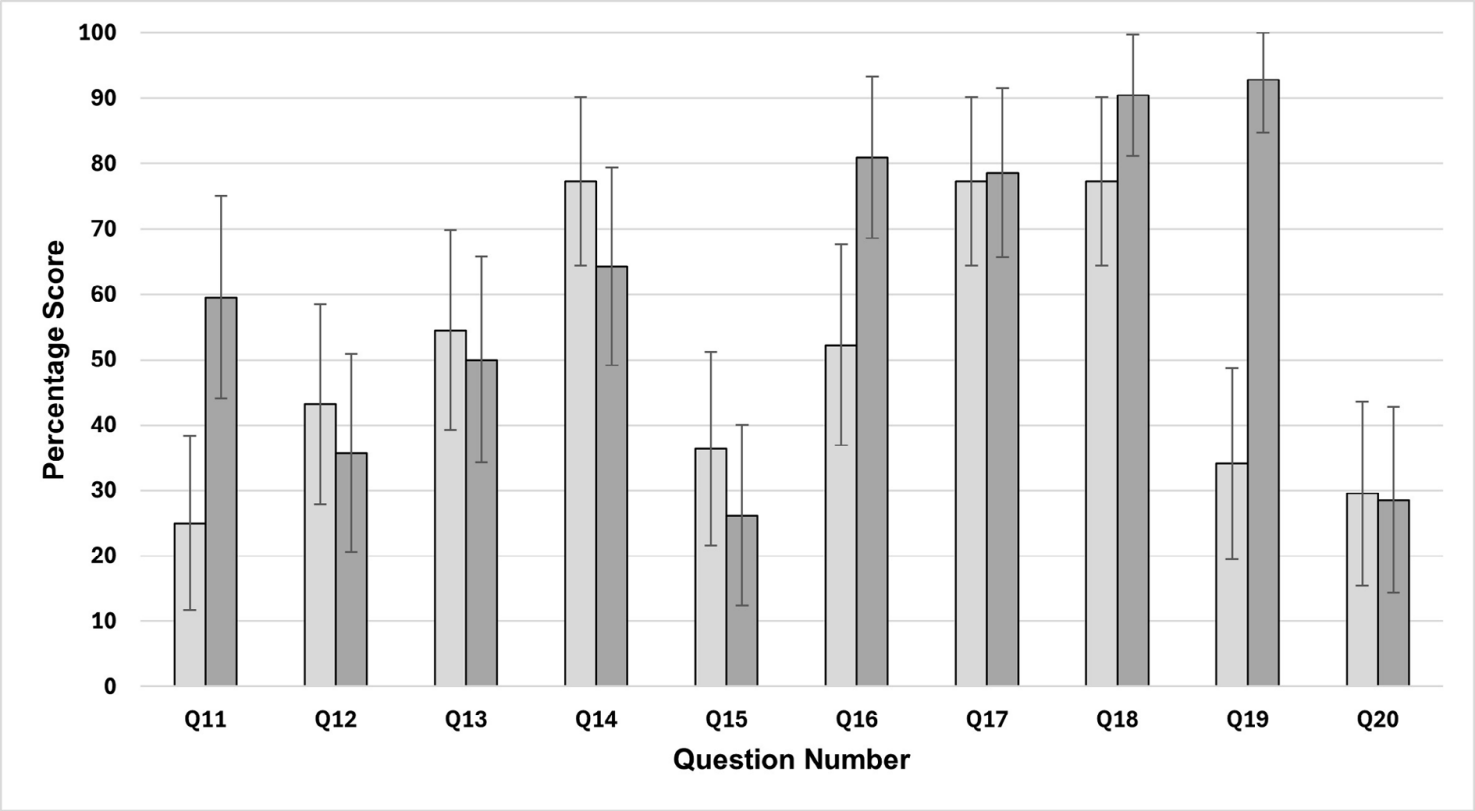
Differences in individual MCQ post scores (mean and 95% confidence interval). The control group is the light grey bars (n = 44), the intervention group is the darker grey bars (n = 42).

The average individual MCQ post scores for the two groups are shown in Figure 3.

## Discussion

This study shows that attending a small group clinical teaching and CBL session improves student knowledge of the diagnosis and management of accommodation and convergence anomalies. To the research team’s knowledge, this was the first study of its kind in optometric education in the UK. The uniqueness of the current study lies in the evaluation approach which randomised the assessment of student learning rather than the instructional delivery. Randomising instructional delivery could have compromised the information students received, rendering it inconsistent and possibly affecting their upcoming examination performance.

In the current study, as there was no statistically significant difference between the intervention and control group’s set A (pre-session) MCQ scores, this indicates that students’ knowledge of accommodation and convergence anomalies was similar before the small group teaching session. However, the statistically significant improvement in the intervention group’s set B (post-session) MCQ scores indicates an improvement in their knowledge. This cannot be down to random chance, as the control group’s set B MCQ scores, which were completed pre-session, did not significantly differ from their set A scores. It is presumed that the increase in knowledge after the small group teaching CBL session of the intervention group is attributable to the design of the teaching session. As the first study of its kind, it provides valuable insights into the advantages of small group teaching combined with CBL for optometry undergraduate teaching. This teaching methodology can easily be applied to the teaching of other optometric clinical techniques.

There are few studies which involve evaluation of pre- and post-test scores relating to small group teaching. Singh *et al*. (2016) found small group teaching to be associated with a greater learning gain than traditional lectures for medical students. Small group teaching has also been shown to improve MCQ scores for medical students (Padugupati *et al*., 2017; Vasudevan *et al*., 2024); in these studies, the same MCQs were used pre- and post-test intervention.

The findings of the current study are in line with existing research which has investigated the impact of CBL across different healthcare professions. Shigli *et al*. (2017) investigated the usefulness of CBL in enhancing dental students’ knowledge. The post-test scores from the CBL group were significantly higher than the pre-test scores (p < 0.01). Here the investigators used the same questions for the pre- and post-test. Said *et al*. (2020) used CBL with dermatology residents. The results showed that the post-test knowledge scores were significantly higher than the pre-test scores (p < 0.01). The full set of MCQs used is not published however the authors stated there were ‘15 content-related questions of comparable difficulty’ (Said *et al*., 2020, p. 405). Foutch, Awad-Amani and Conner (2022) also found that the post-test scores in the CBL group were significantly higher than the pre-test scores (p < 0.01) when investigating CBL versus traditional lectures in a public health course for optometry students. Porter and Parmar (2025) have shown CBL to be an effective way of delivering an Interprofessional Education workshop for pharmacy and optometry students, with MCQ scores increasing after the teaching session.

Looking closer at the current study’s findings, individual differences in the post-session MCQ scores for both the control and intervention groups suggested students generally struggled to answer the following questions which had a mean score of <50%:

- Question 12: An emmetropic orthophoric patient with a normal AC/A ratio has –2.00DS lenses inserted into the Maddox Wing they are looking through. What is the expected result?
- Question 15: What is the normal amplitude of accommodation for a 36-year-old?
- Question 20: You accidentally measure accommodation in a 15-year-old +5.00DS hyperope without their spectacles on, which of the following values is expected?

These results are surprising because the topic of accommodation is taught in several modules before undergraduate optometry students reach their final year at The University of Manchester: Optometric Examination (first and second year), Visual Optics (second year) and Binocular Vision (second year). Final year students would also routinely measure amplitude of accommodation as part of their routine eye examinations. It is possible the use of open book exams in some of these modules has allowed students to progress without properly consolidating their knowledge.

The intervention group performed better than the control group on the following post-session questions:

- Question 11: What is the normal value for accommodative facility?
- Question 16: Which clinical sign is typically associated with accommodative insufficiency?
- Question 19: Which of the following is the first line of treatment for accommodative infacility?

Accommodative facility was one of the techniques taught in the practical sessions; the diagnosis and management of accommodative infacility was covered in the group discussion at the end. This session would have been the first-time students consciously linked clinical techniques with patient results. The case-based learning part of the tutorial involved a patient with accommodative insufficiency. The answers to these questions would have been discussed by the students in their small groups and then together with the instructors at the end of the session. It is possible the discussion with the instructors played a part in the gain in knowledge. However, the students were asked to give their answers to the questions and the instructors only cleared any misunderstanding, therefore the ‘teaching’ they delivered was minimal.

### Strengths/Limitations

This research exhibited several notable strengths. Rather than randomising the educational materials or the delivery provided to students which could compromise student understanding and examination outcomes, the assessment itself was randomised. There was a substantial participation rate and completeness of data sets. Many students who took part in the session had full data sets available for analysis: 86 (97%) final-year undergraduate optometry students. All students received identical teaching from the same two academic supervisors despite being divided into two groups.

Whilst this research provides valuable insights, it has some limitations. This was carried out in a Russell Group university in the United Kingdom and as a result the research may not be generalisable to other UK or international institutions. There was one typographical error in the intervention groups’ post-session questions: the word ‘insufficiency’ was used instead of ‘infacility’ (in the context of managing an accommodation anomaly). However, the management of both these conditions is the same, therefore this error is unlikely to have significantly impacted the overall results. The students in the 23/24 academic year had their education negatively impacted by the COVID-19 pandemic. In their first year, open book exams were used so it is possible the consolidation of their knowledge was not as it would have been for cohorts outside the pandemic. Our study did not randomise the students by academic ability, however the fact there was no difference in the set A results before the teaching started confirms the students’ knowledge was similar at the start of the small group teaching session. Other demographic data such as ethnicity, disability and gender, which were not collected in the current study, are also known to affect student outcomes (Bolton and Lewis, 2024).

Future work could address if the students who are academically weaker gain more from the small group teaching sessions. It would also be useful to gain qualitative data on how students perceive the sessions as well as investigating if this effect occurs across the other modules in optometry.

## Conclusion

To the research team’s knowledge, this is the first study to investigate the impact of small group clinical teaching combined with CBL in optometry education. The study demonstrated that this teaching methodology can enhance undergraduate optometry students’ knowledge of the diagnosis and management of accommodative and convergence anomalies. These findings confirm that small group clinical teaching and CBL are as effective in optometry education as it is in the other areas of health professionals’ undergraduate education.

## Additional File

The additional file for this article can be found as follows:

10.22599/bioj.486.s1 Appendices. Appendix 1 and 2.
