# Supplementary material for: Exploring the Impact of Small Group Teaching and Case-Based Learning on Optometry Students’ Binocular Vision Clinical Knowledge
Source: Br Ir Orthopt J. 2025 Aug 25;21(1):88–94. doi: 10.22599/bioj.486 (PMC12396193; doi:10.22599/bioj.486)
Supplement: Appendices. — Appendix 1 and 2. [file bioj-21-1-486-s1.pdf]

Appendix 1: Teaching materials

Tutorial 2 - Accommodation and Convergence (CC 8.2.4)

These two functions are particularly important to optometrists. Too many children have difficulty in school because these basic functions have not been investigated properly.

In the routine eye examination accommodation is measured using the push up/pull down method and the average of these is taken. It is important you pay attention to both the monocular and binocular readings. If the binocular amplitude of accommodation (AofA) reading is lower than the monocular value, your patient **HAS A BV PROBLEM**.

If the patient is very young you may need to use a -4.00DS lens to get a more accurate reading. Remember to subtract this to any value you find. E.g. 20 – (-4) = 24

Age	Expected Amplitude of Accommodation (D)
5	
10	
20	
30	
40	

Amplitude of Accommodation (RAF Rule)

Measure and record this in the table below.

Value	RE (D)	LE (D)	Binoc (D)
**Push Up**			
**Pull Down**			
**Average**			

Other ways to investigate accommodation

- Monocular Estimate Method (MEM) Retinoscopy
- Accommodative facility (using +/- 2.00DS flippers)

MEM Retinoscopy

This assesses accommodative accuracy. Large lags (> +0.75DS/ +1.00DS) or any lead of accommodation at near are abnormal and indicative of problems.

Method

- Dim the room lights.
- With the patient's distance prescription in place ask them to fix a detailed target on the retinoscope at 40cm.
- (This is what those little cards with words on are for in the Keeler/Heine sets) otherwise use a budgie stick and **hold it at the same distance** as the retinoscope lamp.
- With the ret beam horizontal sweep it across one eye only (this may be different in the paediatric clinic)
- You should see a quick with movement, using plus lenses (held in place for a fraction of a second) neutralise the movement
- Repeat on second eye and record your results

Patient	Result RE	Result LE	Interpretation

Questions

What would it mean if a value of > +1.00DS was found?

What would it mean if a negative value was found?

Accommodative Facility

This is a measure of how quickly we can change our focus from distance to near. You will measure this monocularly and binocularly. **MAKE SURE YOU COVER THE EYE PROPERLY.**

Method

- Hold small (N6) size print (preferably numbers) at 40 cm
- With the patient's distance correction in place, explain you wish them to read the numbers aloud
- Explain it may take a moment for them to come into focus

- You are going to time how may ‘flips’ they do in 1 minute so have a watch with second hands ready
- Place the lenses in front of the patient’s eyes and get them to start reading
- When the row is cleared flip the lenses to the other side
- 1 cycle is a change from plus to minus and back to plus again
- Record how many cpm
- Note if a patient has particular difficulty clearing either plus or minus lenses
- Zellers *et al* (1984) found the normal response was 7.7 cpm binocularly and 11cpm monocularly

In the table below record the results of accommodative facility for all those in your group.

**Patient**	**Monocular Accommodative Facility**		**Binocular Accommodative Facility**
	**RE**	**LE**	

Questions

What would it mean if a patient found it difficult to clear the plus lenses?
What would it mean if a patient found it difficult to clear the minus lenses?
What exercises could you give to help improve accommodation problems?
What problems would a patient with accommodative infacility complain of?
What is accommodative insufficiency?
How does accommodative insufficiency differ from presbyopia?

Convergence

In the table below record your convergence for all those in your group.

**Patient**	**Jump Convergence (step)**	**Near Point of Convergence (NPC) (pursuit)**

When might you record and objective vs a subjective measurement of NPC?

What symptoms might someone with a poor NPC complain of?

What exercises can the optometrist use to improve convergence?

Case Record 1 – Investigation and management of accommodation problems

History and Symptoms

**Name**	Master LH	**Age**	15		
**RFV**	Blurred vision at near				
**DV**	Blurred	**IV**	Blurred	**NV**	Blurs after 1 hour in school
**GH**	Good	**Meds**	None	**Allergies**	None
**OH**	nil	**H/A**	Dull frontal ache starts after 1 hour of close work<br>Has been happening for 4/52	**Dip**	none
**F/F**	None	**FOH**	Mum and dad myopic early teenage years	FGH	Gran Type II diabetes
**Hobbies**	X Box<br>Football				

Examination

**Visions**	**RE**	**LE**	**Binoc**
**DV**	6/5	6/5	6/5
**NV (33cm)**	N5	N5	N5

Prescription Details

**RE**	+0.50 DS
**LE**	+0.50 DS

BV

**Cover Test with and without Rx**	Distance: NMD<br>Near: 6^ XOP good recovery
**Ocular Motility**	Full smooth and accurate
**A of A**	RE: 8D LE: 8D Binoc: 7.5 D (with effort)
**Accommodative Lag**	+2.00 right and left
**Accommodative facility**	3 cpm (cannot clear minus)
**NPC**	8 cm
**Stereopsis**	60” TNO

Ocular health – Nil of note

Questions?

Would any other tests be useful to help you make a diagnosis?

Which results are normal and why?

Which results are abnormal and why?

What is your diagnosis?

How have you reached this conclusion?

What is your management?

What are the management options available for accommodation and convergence issues?

When would you see the child again?

Exercises for accommodation and convergence problems

(make any notes you need to here)

Appendix 2: Sets of 20 multiple-choice questions (MCQs)

Question Number	Set A/ B	Question
**1**	**A**	A person with accommodative spasm may:<br>• Seem more myopic than they really are<br>• Require cycloplegic refraction<br>• Have a lag of accommodation on MEM retinoscopy<br>• Seem more hyperopic than they really are<br>• Have a lead of accommodation on MEM retinoscopy
**2**	**A**	An emmetropic orthophoric patient with a normal AC/A ratio has +2.00DS lenses inserted into the Maddox Wing they are looking through. What is the expected result?<br>• 8^ Esophoria<br>• 10^ Esophoria<br>• 10^ Exophoria<br>• 8^ Exophoria<br>• orthophoria
**3**	**A**	The following diagram represents what a patient may see when using a dot card.<br>Which of the following statements is correct about the diagram?<br>• The patient is using binocular single vision to fuse the 2nd dot, and only sees uncrossed diplopia of the lines behind the dot<br>• The patient is using binocular single vision to fuse the 2nd dot, and only sees crossed diplopia of the lines in front of the dot<br>• The patient is using binocular single vision to fuse the 2nd dot, and sees uncrossed diplopia of the lines behind the dot as well as crossed diplopia of the lines in front of the dot<br>• The patient is using binocular single vision to fuse the 2nd dot, and sees crossed diplopia of the lines behind the dot as well as uncrossed diplopia of the lines in front of the dot<br>• The patient sees pathological diplopia of the line and dots
**4**	**A**	What is the dioptric stimulus to accommodate for an object located at a distance of 25 cm?<br>• 4D<br>• 2.5D<br>• 3.5D

		• 5D<br>• 3D
**5**	**A**	What is the normal amplitude of accommodation for a 12-year-old?<br>• 8D<br>• 6D<br>• 10D<br>• 12D<br>• 20D
**6**	**A**	Which clinical sign is typically associated with convergence insufficiency?<br>• NPC greater than 10 cm<br>• An esophoria at near<br>• An esotropia at near<br>• NPC less than 10cm<br>• Higher binocular amplitude of accommodation than monocular
**7**	**A**	Which of the following descriptions defines Amplitude of Accommodation?<br>• The maximum amount of accommodation the eye can exert<br>• The ability of the eye to focus on stimuli at various distances and in different sequences in a given period of time<br>• The amount by which the accommodative response of the eye is less than the dioptric stimulus of accommodation<br>• The amount by which the accommodative response of the eye is greater than the dioptric stimulus of accommodation<br>• The minimum amount of accommodation the eye can exert
**8**	**A**	Which of the following is convergence measurements is classed as convergence insufficiency?<br>• More than 5 cm<br>• More than 10 cm<br>• More than 6 cm<br>• More than 7 cm<br>• More than 8 cm
**9**	**A**	Which of the following is the first line of treatment for accommodative insufficiency?<br>• Low plus lenses<br>• Dot Card<br>• Hart Chart<br>• Base in prism for near only

		• Brock String
**10**	**A**	You accidentally measure accommodation in a 12-year-old +3.00 hyperope without their spectacles on, which of the following values is expected?<br>• 18D<br>• 11D<br>• 9D<br>• 16D<br>• 8D
**11**	**B**	What is the normal value for accommodative facility?<br>• 8 cpm binocularly, 11 cpm monocularly<br>• 8 cpm monocularly, 11 cpm binocularly<br>• 8 cpm binocularly, 5 cpm monocularly<br>• 8 cpm binocularly, 8 cpm monocularly
**12**	**B**	An emmetropic orthophoric patient with a normal AC/A ratio has -2.00DS lenses inserted into the Maddox Wing they are looking through. What is the expected result?<br>• 8^ Esophoria<br>• 10^ Esophoria<br>• 10^ Exophoria<br>• 8^ Exophoria<br>• orthophoria
**13**	**B**	The following diagram represents what a patient may see when using a brock string.<br>Which of the following statements is correct about the diagram?<br>• The patient is using binocular single vision to fuse the green bead, and only sees uncrossed diplopia of the lines behind the bead<br>• The patient is using binocular single vision to fuse the green bead, and only sees crossed diplopia of the lines in front of the bead<br>• The patient is using binocular single vision to fuse the green bead, and sees uncrossed diplopia of the beads behind the green bead as well as crossed diplopia of the lines in front of the green bead<br>• The patient is using binocular single vision to fuse the green bead, and sees crossed diplopia of the lines behind the bead as well as uncrossed diplopia of the lines in front of the bead<br>• The patient sees pathological diplopia of the bead and string
**14**	**B**	What is the dioptric stimulus to accommodate for an object located at

		30cm?<br>• 3D<br>• 3.25D<br>• 3.5D<br>• 3.75D<br>• 2.5D
**15**	**B**	What is the normal amplitude of accommodation for a 36-year-old?<br>• 8D<br>• 7D<br>• 5D<br>• 4D<br>• 9D
**16**	**B**	Which clinical sign is typically associated with accommodative insufficiency?<br>• Accommodative lag of +0.75DS<br>• Binocular accommodative facility of 12cpm<br>• Accommodative lead of -1.00DS<br>• NPC of 5 cm<br>• Accommodative lag of +1.75DS
**17**	**B**	Which of the following descriptions defines accommodative lag?<br>• The maximum amount of accommodation the eye can exert<br>• The ability of the eye to focus on stimuli at various distances and in different sequences in a given period of time<br>• The amount by which the accommodative response of the eye is less than the dioptric stimulus of accommodation<br>• The amount by which the accommodative response of the eye is greater than the dioptric stimulus of accommodation<br>• The minimum amount of accommodation the eye can exert
**18**	**B**	Which of the following is a normal near point of convergence?<br>• Less than 5 cm<br>• Less than 10 cm<br>• Less than 12 cm<br>• Less than 6 cm<br>• Less than 15 cm
**19**	**B**	Which of the following is the first line of treatment for accommodative infacility?

		• Low plus lenses<br>• Dot Card<br>• Hart Chart<br>• Base in prism for near only<br>• Brock String
**20**	**B**	You accidentally measure accommodation in a 15-year-old +5.00 hyperope without their spectacles on, which of the following values is expected?<br>• 18D<br>• 10D<br>• 12D<br>• 8D<br>• 9D
